# Crystal structure of 1,4,5,6,7,8,9,10,11,12,13-undeca­hydro­cyclo­dodeca[*c*]pyrazol-3-ol

**DOI:** 10.1107/S2056989015016746

**Published:** 2015-09-12

**Authors:** Casey C. Raymond, Michael A. Knopp

**Affiliations:** a Department of Chemistry, 296 Shineman Center, SUNY Oswego, Oswego, NY 13126, USA; b Environmental Science and Sustainability Department, 307 South Hall, 181 Main St., University of Maine at Presque Isle, Presque Isle, Maine 04769, USA

**Keywords:** crystal structure, pyrazolol, tautomer, pyrazolone, macrocycle, O—H⋯N hydrogen bond, N—H⋯π inter­action

## Abstract

The title compound, C_13_H_22_N_2_O, crystallized as a pyrazolol tautomer. The 12-membered macrocycle has a distorted chair conformation. In the crystal, mol­ecules are linked *via* pairs of O—H⋯N hydrogen bonds, forming inversion dimers. The dimers are linked *via* N—H⋯π and C—H⋯π inter­actions, forming slabs parallel to the *bc* plane.

## Related literature   

The crystal structure of the title compound clarifies the connectivity of a class of pyrazolone-derived materials, specifically revealing a pyrazolol tautomer instead of the expected pyrazolone. For the synthesis of the title compound, see: Silveira *et al.* (1977 ▸). For the structure of a similar tautomer, see: Silveira *et al.* (1980 ▸). For a review of the chemistry of pyrazolo­nes, pyrazolidones and their derivatives, see: Wiley & Wiley (1964 ▸).
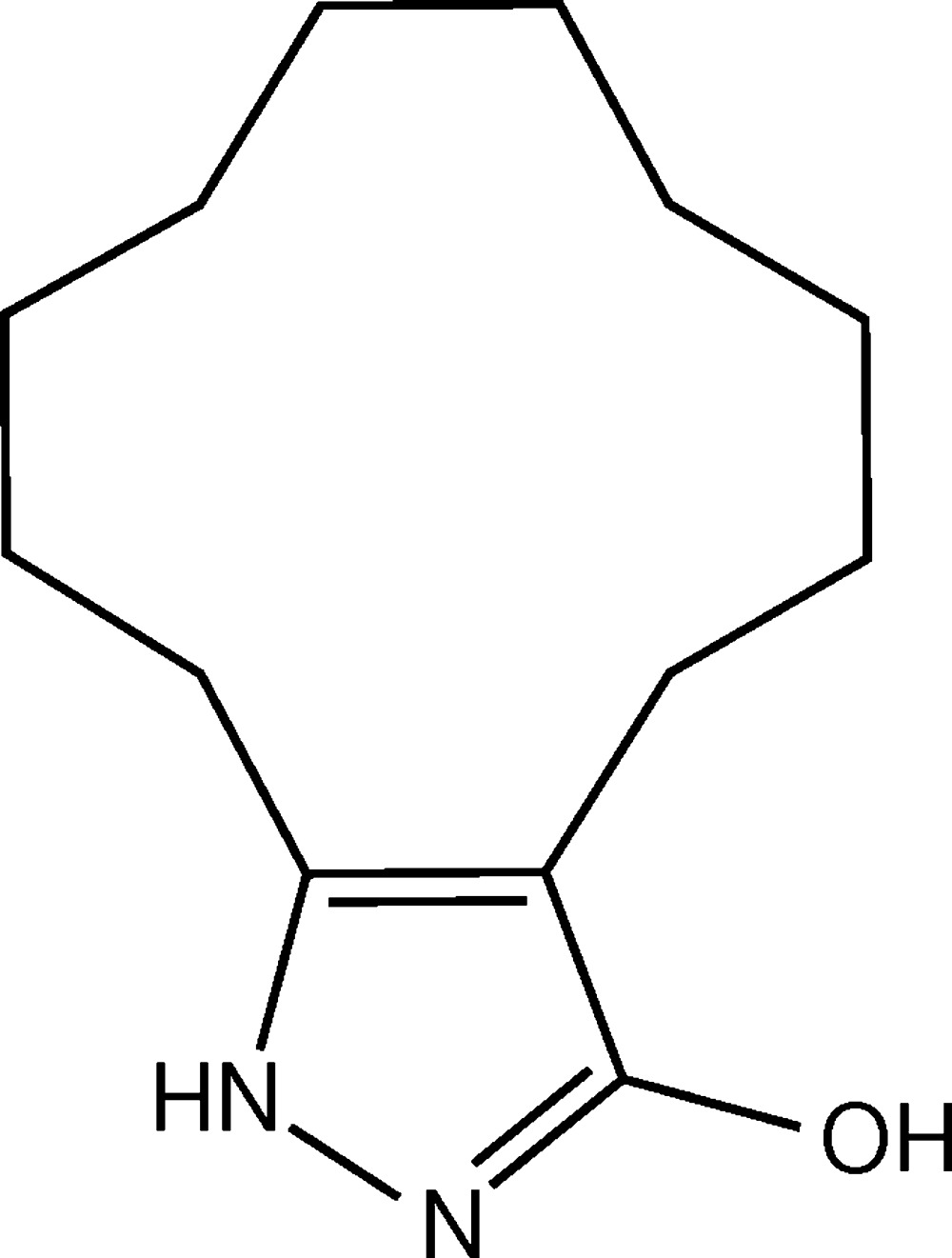


## Experimental   

### Crystal data  

C_13_H_22_N_2_O
*M* *_r_* = 222.32
Monoclinic, 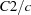

*a* = 30.008 (2) Å
*b* = 7.4764 (5) Å
*c* = 11.6516 (8) Å
β = 105.4374 (12)°
*V* = 2519.7 (3) Å^3^
*Z* = 8
Mo *K*α radiation
μ = 0.08 mm^−1^
*T* = 100 K
0.59 × 0.33 × 0.11 mm

### Data collection  

Bruker APEX CCD diffractometer
Absorption correction: multi-scan (*SADABS*; Bruker, 2007 ▸) *T*_min_ = 0.871, *T*_max_ = 0.992
14420 measured reflections
3845 independent reflections
3383 reflections with *I* > 2σ(*I*)
*R*_int_ = 0.041

### Refinement  

*R*[*F* ^2^ > 2σ(*F* ^2^)] = 0.051
*wR*(*F* ^2^) = 0.119
*S* = 1.03
3845 reflections
146 parameters
H-atom parameters constrained
Δρ_max_ = 0.41 e Å^−3^
Δρ_min_ = −0.20 e Å^−3^

### 

Data collection: *APEX2* (Bruker, 2007 ▸); cell refinement: *SAINT* (Bruker, 2007 ▸); data reduction: *SAINT*; program(s) used to solve structure: *SHELXS97* (Sheldrick, 2008 ▸); program(s) used to refine structure: *SHELXL2014* (Sheldrick, 2015 ▸); molecular graphics: *Mercury* (Macrae *et al.*, 2008 ▸); software used to prepare material for publication: *SHELXTL* (Sheldrick, 2008 ▸).

## Supplementary Material

Crystal structure: contains datablock(s) I, New_Global_Publ_Block. DOI: 10.1107/S2056989015016746/su5204sup1.cif

Structure factors: contains datablock(s) I. DOI: 10.1107/S2056989015016746/su5204Isup2.hkl

Supporting information file. DOI: 10.1107/S2056989015016746/su5204Isup3.cdx

Supporting information file. DOI: 10.1107/S2056989015016746/su5204Isup4.cml

**Fig. 1.**
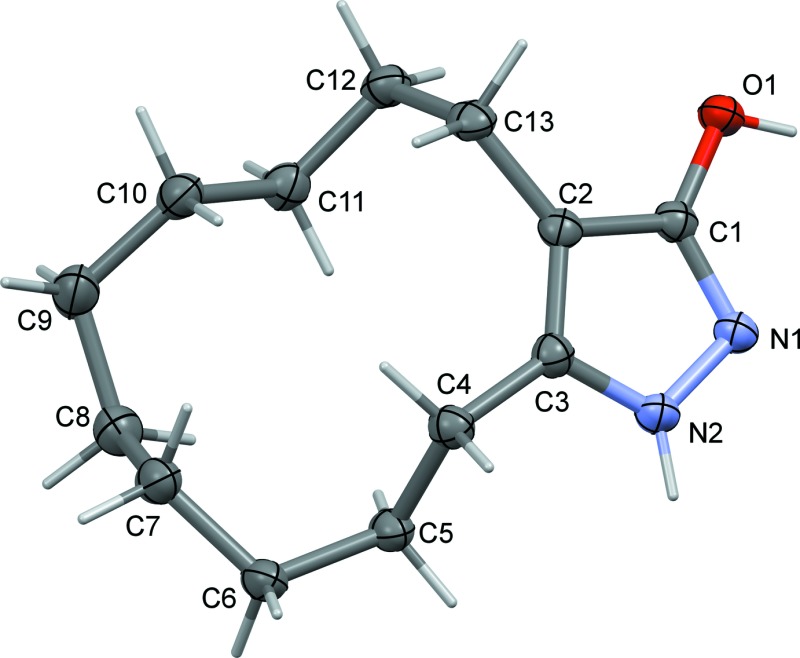
A view of the molecular structure of the title compound, with atom labeling. Displacement ellipsoids are drawn at the 50% probability level.

**Fig. 2.**
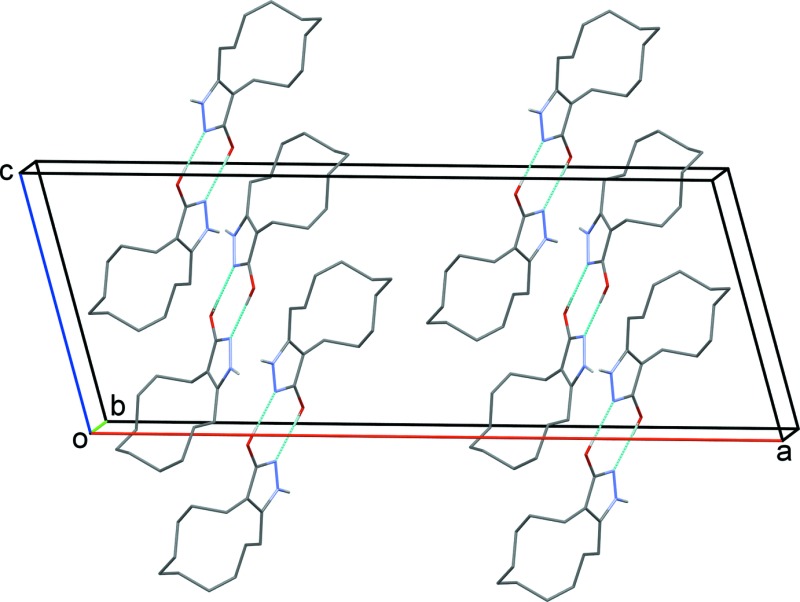
A view along the b axis of the crystal packing of the title compound. Hydrogen bonds are shown as dashed lines (see Table 1), and C-bound H atoms have been omitted for clarity.

. DOI: 10.1107/S2056989015016746/su5204fig1.tif
A view of the mol­ecular structure of the title compound, with atom labeling. Displacement ellipsoids are drawn at the 50% probability level.

b . DOI: 10.1107/S2056989015016746/su5204fig2.tif
A view along the *b* axis of the crystal packing of the title compound. Hydrogen bonds are shown as dashed lines (see Table 1), and C-bound H atoms have been omitted for clarity.

CCDC reference: 1422925

Additional supporting information:  crystallographic information; 3D view; checkCIF report

## Figures and Tables

**Table 1:** Hydrogen-bond geometry (, ) *Cg* is the centroid of the pyrazol ring N1/N2/C1C3.

*D*H*A*	*D*H	H*A*	*D* *A*	*D*H*A*
O1H1N1^i^	0.84	1.87	2.7072(12)	177
N2H2*Cg* ^ii^	0.88	2.58	3.4429(11)	166
C6H6*B* *Cg* ^iii^	0.99	2.71	3.5734(13)	147

Symmetry codes: (i) 
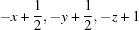
; (ii) 
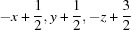
; (iii) 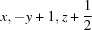.

## supplementary crystallographic information

### S1. Comment

The crystal structure of the title compound, Fig. 1, clarifies the connectivity of a class of pyrazolone-derived materials, specifically revealing a pyrzazolol tautomer instead of the expected pyrazolone. The bond lengths and angles support this pyrazolol tautomer in the solid state. The crystal structure reveals a different isomer than originally proposed by by (Silveira *et al.*, 1977). However, it is similar to a tautomer proposed in a subsequent investigation (Silveira *et al.*, 1980).

Specifically, we have determined that the compound of inter­est contains an alcohol group instead of the postulated ketone. The alcoholic group is also consistent with a positive reaction with iron(III) solutions, yielding a dark purple color (Wiley & Wiley, 1964).

In the crystal, molecules are linked via a pair of O—H···N hydrogen bonds forming inversion dimers (Fig. 2 and Table 1). The dimers are linked via N—H···π and C—H···π inter­actions forming slabs parallel to the bc plane (Table 1).

### S2. Synthesis and crystallization

The title compound was prepared as described by (Silveira *et al.*, 1977). Colorless crystals were obtained by recrystallization by slow cooling of a saturated, warm ethanol solution.

### S3. Refinement details

The NH and OH H atoms were located in a difference Fourier map. All of the H atoms were placed in calculated positions and refined as riding: O—H = 0.84 Å, N—H = 0.88 Å, C—H = 0.99 Å with U_iso_(H) = 1.5U_eq_(O) for the OH H atom and 1.2U_eq_(N,C) for other H atoms.

### Crystal data

**Table:**

C_13_H_22_N_2_O	*F*(000) = 976
*M**_r_* = 222.32	*D*_x_ = 1.172 Mg m^−^^3^
Monoclinic, *C*2/*c*	Mo *K*α radiation, λ = 0.71073 Å
*a* = 30.008 (2) Å	Cell parameters from 9304 reflections
*b* = 7.4764 (5) Å	θ = 2.8–30.5°
*c* = 11.6516 (8) Å	µ = 0.08 mm^−^^1^
β = 105.4374 (12)°	*T* = 100 K
*V* = 2519.7 (3) Å^3^	Plate, colorless
*Z* = 8	0.59 × 0.33 × 0.11 mm

### Data collection

**Table:**

Bruker APEX CCD diffractometer	3383 reflections with *I* > 2σ(*I*)
ω and phi scans	*R*_int_ = 0.041
Absorption correction: multi-scan (*SADABS*; Bruker, 2007)	θ_max_ = 30.5°, θ_min_ = 2.8°
*T*_min_ = 0.871, *T*_max_ = 0.992	*h* = −42→42
14420 measured reflections	*k* = −10→10
3845 independent reflections	*l* = −16→16

### Refinement

**Table:**

Refinement on *F*^2^	0 restraints
Least-squares matrix: full	Hydrogen site location: inferred from neighbouring sites
*R*[*F*^2^ > 2σ(*F*^2^)] = 0.051	H-atom parameters constrained
*wR*(*F*^2^) = 0.119	*w* = 1/[σ^2^(*F*_o_^2^) + (0.0437*P*)^2^ + 2.2017*P*] where *P* = (*F*_o_^2^ + 2*F*_c_^2^)/3
*S* = 1.03	(Δ/σ)_max_ = 0.001
3845 reflections	Δρ_max_ = 0.41 e Å^−^^3^
146 parameters	Δρ_min_ = −0.20 e Å^−^^3^

### Special details

**Table:**

Geometry. All e.s.d.'s (except the e.s.d. in the dihedral angle between two l.s. planes) are estimated using the full covariance matrix. The cell e.s.d.'s are taken into account individually in the estimation of e.s.d.'s in distances, angles and torsion angles; correlations between e.s.d.'s in cell parameters are only used when they are defined by crystal symmetry. An approximate (isotropic) treatment of cell e.s.d.'s is used for estimating e.s.d.'s involving l.s. planes.

### Fractional atomic coordinates and isotropic or equivalent isotropic displacement parameters (Å^2^)

**Table:**

	*x*	*y*	*z*	*U*_iso_*/*U*_eq_	
O1	0.29213 (3)	0.05291 (11)	0.56411 (7)	0.02556 (18)	
H1	0.2743	0.0908	0.5005	0.038*	
N1	0.26539 (3)	0.31282 (13)	0.63710 (8)	0.0237 (2)	
N2	0.27474 (3)	0.38692 (13)	0.74851 (8)	0.0249 (2)	
H2	0.2628	0.4887	0.7641	0.030*	
C1	0.29108 (4)	0.16479 (14)	0.65347 (9)	0.0214 (2)	
C2	0.31597 (4)	0.13945 (14)	0.77385 (9)	0.0208 (2)	
C3	0.30426 (4)	0.28695 (15)	0.83204 (9)	0.0221 (2)	
C4	0.32060 (4)	0.34607 (15)	0.95885 (9)	0.0240 (2)	
H4A	0.3412	0.2533	1.0056	0.029*	
H4B	0.2937	0.3591	0.9922	0.029*	
C5	0.34679 (4)	0.52462 (15)	0.97093 (10)	0.0258 (2)	
H5A	0.3241	0.6224	0.9444	0.031*	
H5B	0.3674	0.5230	0.9171	0.031*	
C6	0.37587 (4)	0.56546 (16)	1.09762 (10)	0.0284 (2)	
H6A	0.3889	0.6874	1.0991	0.034*	
H6B	0.3554	0.5647	1.1517	0.034*	
C7	0.41558 (4)	0.43390 (17)	1.14525 (10)	0.0296 (2)	
H7A	0.4026	0.3117	1.1419	0.036*	
H7B	0.4302	0.4623	1.2299	0.036*	
C8	0.45307 (4)	0.43498 (18)	1.07776 (12)	0.0336 (3)	
H8A	0.4392	0.4770	0.9954	0.040*	
H8B	0.4774	0.5215	1.1165	0.040*	
C9	0.47548 (4)	0.2527 (2)	1.07272 (12)	0.0363 (3)	
H9A	0.4882	0.2088	1.1551	0.044*	
H9B	0.5017	0.2682	1.0370	0.044*	
C10	0.44297 (4)	0.10995 (17)	1.00175 (10)	0.0291 (2)	
H10A	0.4592	−0.0065	1.0134	0.035*	
H10B	0.4159	0.0992	1.0346	0.035*	
C11	0.42583 (4)	0.14687 (16)	0.86845 (10)	0.0263 (2)	
H11A	0.4529	0.1628	0.8359	0.032*	
H11B	0.4082	0.2603	0.8563	0.032*	
C12	0.39528 (4)	−0.00152 (16)	0.79927 (10)	0.0266 (2)	
H12A	0.4114	−0.1172	0.8203	0.032*	
H12B	0.3913	0.0185	0.7131	0.032*	
C13	0.34726 (4)	−0.01540 (15)	0.82185 (10)	0.0248 (2)	
H13A	0.3323	−0.1270	0.7850	0.030*	
H13B	0.3511	−0.0241	0.9087	0.030*	

### Atomic displacement parameters (Å^2^)

**Table:**

	*U*^11^	*U*^22^	*U*^33^	*U*^12^	*U*^13^	*U*^23^
O1	0.0306 (4)	0.0225 (4)	0.0221 (4)	0.0039 (3)	0.0044 (3)	−0.0037 (3)
N1	0.0235 (4)	0.0246 (5)	0.0222 (4)	0.0022 (3)	0.0048 (3)	−0.0047 (3)
N2	0.0259 (4)	0.0248 (5)	0.0227 (4)	0.0054 (4)	0.0044 (3)	−0.0055 (4)
C1	0.0209 (4)	0.0205 (5)	0.0238 (5)	−0.0019 (4)	0.0076 (4)	−0.0018 (4)
C2	0.0222 (4)	0.0189 (5)	0.0219 (5)	−0.0015 (4)	0.0069 (4)	0.0006 (4)
C3	0.0210 (4)	0.0230 (5)	0.0225 (5)	−0.0004 (4)	0.0059 (4)	−0.0001 (4)
C4	0.0286 (5)	0.0230 (5)	0.0207 (5)	0.0016 (4)	0.0069 (4)	−0.0010 (4)
C5	0.0313 (5)	0.0210 (5)	0.0227 (5)	0.0025 (4)	0.0028 (4)	−0.0006 (4)
C6	0.0342 (6)	0.0238 (5)	0.0242 (5)	0.0046 (4)	0.0023 (4)	−0.0052 (4)
C7	0.0342 (6)	0.0298 (6)	0.0220 (5)	0.0067 (5)	0.0026 (4)	−0.0025 (4)
C8	0.0302 (6)	0.0344 (7)	0.0338 (6)	−0.0005 (5)	0.0045 (5)	−0.0064 (5)
C9	0.0291 (6)	0.0442 (8)	0.0317 (6)	0.0084 (5)	0.0013 (5)	−0.0069 (5)
C10	0.0312 (6)	0.0311 (6)	0.0236 (5)	0.0100 (5)	0.0050 (4)	−0.0001 (4)
C11	0.0258 (5)	0.0301 (6)	0.0238 (5)	0.0033 (4)	0.0080 (4)	0.0005 (4)
C12	0.0311 (5)	0.0260 (5)	0.0229 (5)	0.0076 (4)	0.0076 (4)	−0.0010 (4)
C13	0.0312 (5)	0.0176 (5)	0.0254 (5)	0.0011 (4)	0.0072 (4)	0.0015 (4)

### Geometric parameters (Å, º)

**Table:**

O1—C1	1.3424 (13)	C7—H7A	0.9900
O1—H1	0.8400	C7—H7B	0.9900
N1—C1	1.3331 (14)	C8—C9	1.5280 (19)
N1—N2	1.3700 (12)	C8—H8A	0.9900
N2—C3	1.3533 (14)	C8—H8B	0.9900
N2—H2	0.8800	C9—C10	1.5312 (18)
C1—C2	1.4153 (14)	C9—H9A	0.9900
C2—C3	1.3879 (15)	C9—H9B	0.9900
C2—C13	1.5018 (15)	C10—C11	1.5257 (16)
C3—C4	1.4944 (15)	C10—H10A	0.9900
C4—C5	1.5364 (16)	C10—H10B	0.9900
C4—H4A	0.9900	C11—C12	1.5257 (17)
C4—H4B	0.9900	C11—H11A	0.9900
C5—C6	1.5322 (16)	C11—H11B	0.9900
C5—H5A	0.9900	C12—C13	1.5355 (16)
C5—H5B	0.9900	C12—H12A	0.9900
C6—C7	1.5309 (16)	C12—H12B	0.9900
C6—H6A	0.9900	C13—H13A	0.9900
C6—H6B	0.9900	C13—H13B	0.9900
C7—C8	1.5345 (18)		
			
C1—O1—H1	109.5	H7A—C7—H7B	107.6
C1—N1—N2	103.62 (9)	C9—C8—C7	113.91 (12)
C3—N2—N1	112.81 (9)	C9—C8—H8A	108.8
C3—N2—H2	123.6	C7—C8—H8A	108.8
N1—N2—H2	123.6	C9—C8—H8B	108.8
N1—C1—O1	122.57 (10)	C7—C8—H8B	108.8
N1—C1—C2	112.67 (9)	H8A—C8—H8B	107.7
O1—C1—C2	124.76 (10)	C8—C9—C10	114.75 (10)
C3—C2—C1	104.02 (9)	C8—C9—H9A	108.6
C3—C2—C13	130.18 (10)	C10—C9—H9A	108.6
C1—C2—C13	125.80 (10)	C8—C9—H9B	108.6
N2—C3—C2	106.85 (9)	C10—C9—H9B	108.6
N2—C3—C4	121.83 (10)	H9A—C9—H9B	107.6
C2—C3—C4	131.22 (10)	C11—C10—C9	114.67 (11)
C3—C4—C5	111.89 (9)	C11—C10—H10A	108.6
C3—C4—H4A	109.2	C9—C10—H10A	108.6
C5—C4—H4A	109.2	C11—C10—H10B	108.6
C3—C4—H4B	109.2	C9—C10—H10B	108.6
C5—C4—H4B	109.2	H10A—C10—H10B	107.6
H4A—C4—H4B	107.9	C10—C11—C12	113.49 (10)
C4—C5—C6	114.06 (10)	C10—C11—H11A	108.9
C4—C5—H5A	108.7	C12—C11—H11A	108.9
C6—C5—H5A	108.7	C10—C11—H11B	108.9
C4—C5—H5B	108.7	C12—C11—H11B	108.9
C6—C5—H5B	108.7	H11A—C11—H11B	107.7
H5A—C5—H5B	107.6	C11—C12—C13	114.71 (9)
C7—C6—C5	114.20 (9)	C11—C12—H12A	108.6
C7—C6—H6A	108.7	C13—C12—H12A	108.6
C5—C6—H6A	108.7	C11—C12—H12B	108.6
C7—C6—H6B	108.7	C13—C12—H12B	108.6
C5—C6—H6B	108.7	H12A—C12—H12B	107.6
H6A—C6—H6B	107.6	C2—C13—C12	113.98 (9)
C6—C7—C8	114.64 (10)	C2—C13—H13A	108.8
C6—C7—H7A	108.6	C12—C13—H13A	108.8
C8—C7—H7A	108.6	C2—C13—H13B	108.8
C6—C7—H7B	108.6	C12—C13—H13B	108.8
C8—C7—H7B	108.6	H13A—C13—H13B	107.7
			
C1—N1—N2—C3	−1.49 (12)	N2—C3—C4—C5	−60.38 (14)
N2—N1—C1—O1	−179.34 (10)	C2—C3—C4—C5	115.43 (13)
N2—N1—C1—C2	1.48 (12)	C3—C4—C5—C6	−163.47 (10)
N1—C1—C2—C3	−0.97 (12)	C4—C5—C6—C7	64.21 (14)
O1—C1—C2—C3	179.87 (10)	C5—C6—C7—C8	64.54 (14)
N1—C1—C2—C13	178.86 (10)	C6—C7—C8—C9	−147.20 (11)
O1—C1—C2—C13	−0.30 (17)	C7—C8—C9—C10	65.43 (15)
N1—N2—C3—C2	0.93 (13)	C8—C9—C10—C11	66.47 (15)
N1—N2—C3—C4	177.64 (10)	C9—C10—C11—C12	177.39 (10)
C1—C2—C3—N2	0.01 (11)	C10—C11—C12—C13	70.67 (12)
C13—C2—C3—N2	−179.81 (10)	C3—C2—C13—C12	−101.48 (13)
C1—C2—C3—C4	−176.27 (11)	C1—C2—C13—C12	78.73 (13)
C13—C2—C3—C4	3.91 (19)	C11—C12—C13—C2	68.41 (13)

### Hydrogen-bond geometry (Å, º)

Cg is the centroid of the pyrazol ring N1/N2/C1–C3.

**Table:**

*D*—H···*A*	*D*—H	H···*A*	*D*···*A*	*D*—H···*A*
O1—H1···N1^i^	0.84	1.87	2.7072 (12)	177
N2—H2···*Cg*^ii^	0.88	2.58	3.4429 (11)	166
C6—H6*B*···*Cg*^iii^	0.99	2.71	3.5734 (13)	147

Symmetry codes: (i) −*x*+1/2, −*y*+1/2, −*z*+1; (ii) −*x*+1/2, *y*+1/2, −*z*+3/2; (iii) *x*, −*y*+1, *z*+1/2.
