# Supplementary figures and images for: Crystal structure of 1,4,5,6,7,8,9,10,11,12,13-undeca­hydro­cyclo­dodeca[c]pyrazol-3-ol
Source: Acta Crystallogr E Crystallogr Commun. 2015 Sep 12;71(Pt 10):o752–3. doi: 10.1107/S2056989015016746 (PMC4647396; doi:10.1107/S2056989015016746)

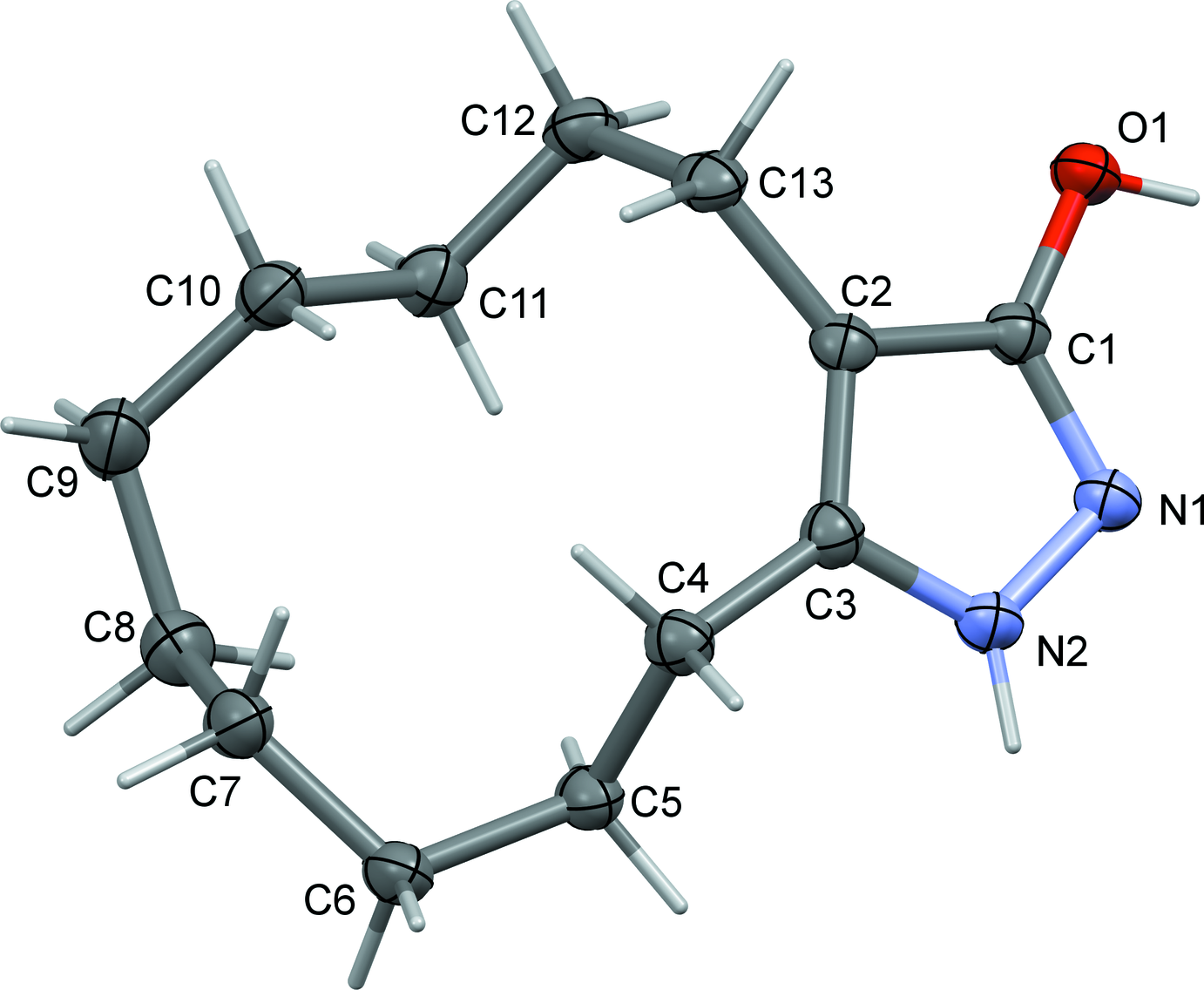

Supplement: Supplementary file 5 [file e-71-0o752-fig1.tif]

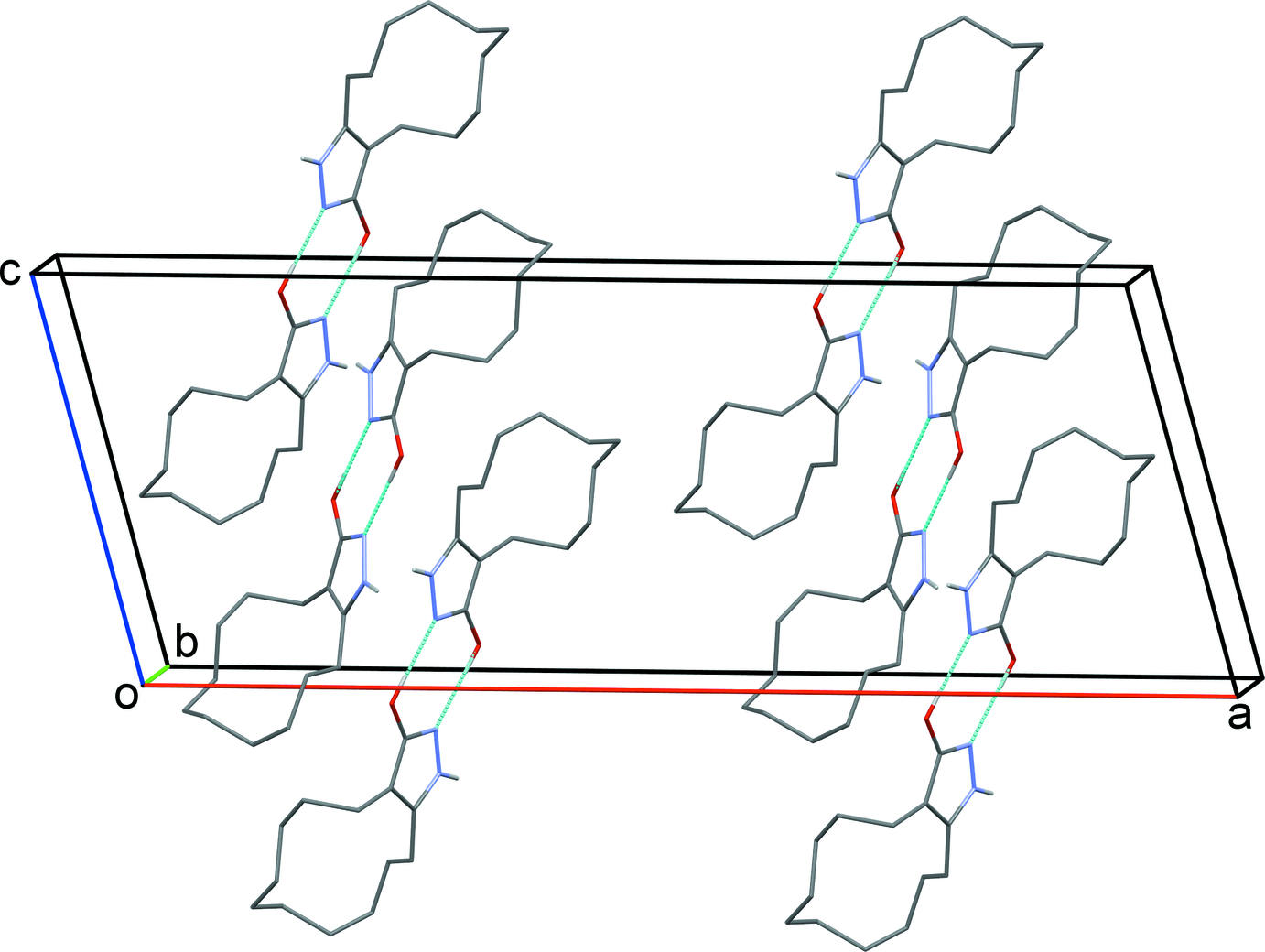

Supplement: Supplementary file 6 [file e-71-0o752-fig2.tif]
